# Current challenges and best practices for cell-free long RNA biomarker discovery

**DOI:** 10.1186/s40364-022-00409-w

**Published:** 2022-08-18

**Authors:** Lluc Cabús, Julien Lagarde, Joao Curado, Esther Lizano, Jennifer Pérez-Boza

**Affiliations:** 1 Institut de Biologia Evolutiva, Universitat Pompeu Fabra, Barcelona, Spain; 2 Flomics Biotech, Barcelona, Spain

**Keywords:** Liquid biopsies, Cell-free RNA, Long RNA, RNA sequencing, Technical bias, Early diagnosis

## Abstract

The analysis of biomarkers in biological fluids, also known as liquid biopsies, is seen with great potential to diagnose complex diseases such as cancer with a high sensitivity and minimal invasiveness. Although it can target any biomolecule, most liquid biopsy studies have focused on circulating nucleic acids. Historically, studies have aimed at the detection of specific mutations on cell-free DNA (cfDNA), but recently, the study of cell-free RNA (cfRNA) has gained traction. Since 2020, a handful of cfDNA tests have been approved for therapy selection by the FDA, however, no cfRNA tests are approved to date. One of the main drawbacks in the field of RNA-based liquid biopsies is the low reproducibility of the results, often caused by technical and biological variability, a lack of standardized protocols and insufficient cohorts. In this review, we will identify the main challenges and biases introduced during the different stages of biomarker discovery in liquid biopsies with cfRNA and propose solutions to minimize them.

## Background

In the past few years, there has been an increased interest in finding minimally invasive methods for disease-specific biomarker detection [1]. Following this trend, liquid biopsies are becoming promising alternatives to replace more invasive diagnostic methods such as tissue biopsies or image-based methods in the future. Although liquid biopsies can theoretically be applied to any biomolecule in any biological fluid, during the last decades there has been an increase in the studies that target circulating nucleic acids in blood [2–5]. Although the discovery and validation of these biomarkers require a considerable economic effort, their implementation in the clinical practice will be cheap, since the process only requires the obtention of a blood sample, but the true economical relief of this method is yet to be determined [6]. Liquid biopsies will constitute a great advantage for early detection, since adherence to the current screening tests are one of the major problems of the healthcare system.

Even though other biofluids are of special interest for certain diseases (urine for prostate or bladder cancer [7, 8], or cerebrospinal fluid for brain diseases, such as Parkinson’s disease or some variants of brain cancer [9, 10]), for the majority of diseases, studies have focused only on blood and the fractions derived from it (plasma, serum and platelets). For this reason, in this review we have focused on circulating biomarkers in blood.

To date, most studies on circulating nucleic acids have centered on diagnosis, prognosis and response to treatment in oncology using cell-free tumor DNA (ctDNA). These are molecules of DNA shed by the tumor and present in the circulation [11]. CtDNA biomarkers provide information about specific mutations and are of special interest for targeted therapy: treatment with drugs directed to those specific mutations present only in the tumor cells [12]. Highlighting their potential, several ctDNA-based screening tests have been approved for the clinical practice during the last years, with many more currently undergoing clinical trials [13]. Although promising, there is one main limitation associated with the translation of ctDNA-based screening tests to the clinic: the abundance of ctDNA in blood is directly linked to tumor burden. CtDNA derives exclusively from tumor cells, and it is secreted into the extracellular milieu either during the processes of cellular death or through active export [14]. These nucleic acids are biologically informative, but present important limitations for early detection in early cancer stages or in diseases with low tumor cells [15, 16]. Unlike ctDNA, cell-free RNA (cfRNA) is released from cancerous and non-cancerous cells. It can derive from non-transformed tissues such as stroma or from the immune system responding to the presence of tumors, both of which can be highly informative for the diagnosis [17].

Changes in RNA expression in cells are a dynamic process that can reflect tissue damage or disease [18]. Moreover, the study of cfRNA is not merely based on the differential abundance of a set of specific genes, but also on additional factors such as pathogenic alternative splicing [19] or A-to-I RNA editing [20], changes that are not detectable in the genome, only in the transcriptome. Due to these limitations, there has been a rising interest in the field of cfRNA over ctDNA in the last few years.

The field of cell-free RNA biomarkers has mostly focused on the study of microRNAs (miRNAs) as biomarkers of disease in the circulation due to their higher stability in blood [21]. However, there is a rising interest in the study of long RNAs (> 200 nt), including but not limited to messenger RNAs (mRNAs) and long non-coding RNAs (lncRNAs). As an example of this, lately some studies have suggested circulating biomarkers based on long RNAs for diseases such as fetal congenital heart defects [22], Alzheimer’s disease [23] and lung cancer [24]. However, none of these have reached the level of validation necessary to reach the clinic yet.

Although more technically challenging, there is one main advantage associated with the study of long RNAs: the number of known long RNAs is much higher than that of known miRNAs (37,911 known long RNA genes between mRNAs and lncRNAs [25] vs 4571 between hairpin and mature miRNA sequences) [26]. This is also the case in biofluids, where the number of mRNAs detected is between 5 and 450 times the number of miRNAs detected [27]. As a result of this higher number of RNAs, the potential to obtain biomarkers that reliably assess the state of a specific disease using these biomolecules is much higher.

**Fig. 1:**
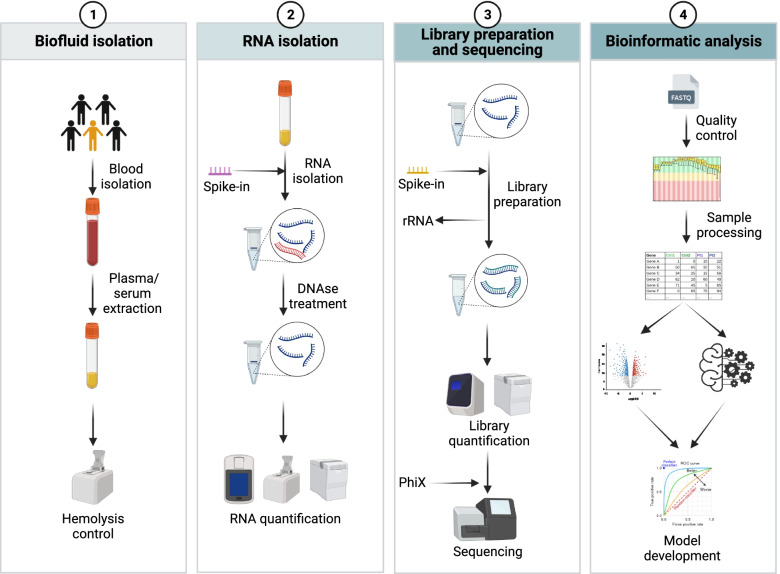
Schematic timeline of all the steps involved in the development of cfRNA biomarkers. (1) Biofluid isolation: after obtaining blood from the patients and centrifugation to get plasma or serum, it is necessary to perform a hemolysis control to measure the contamination by cellular lysis. (2) RNA isolation: prior to the processing of plasma/serum, it is recommended to add external RNA molecules to act as proxies for correct RNA isolation (spike-ins). After isolation of the nucleic acids and before further processing, a step of DNAse digestion is required to limit the contamination of the sample with co-purified DNA. A final step of RNA quantification is required before moving forward to (3) library preparation. To improve reproducibility, a different set of spike-ins are added before starting the process of preparing the RNA for sequencing, and rRNA, lacking biological information, is depleted from the samples. Once the libraries are prepared and quantified, the next step is to sequence them. During this step, it is also possible to add an exogenous library (PhiX), to measure technical variability and ensure reproducibility. (4) Bioinformatic analysis: after the initial quality control of the sample, the data is processed and the expression of several genes linked to a specific phenotype through differential expression analysis or machine learning, to develop a robust biomarker model

**Fig. 2:**
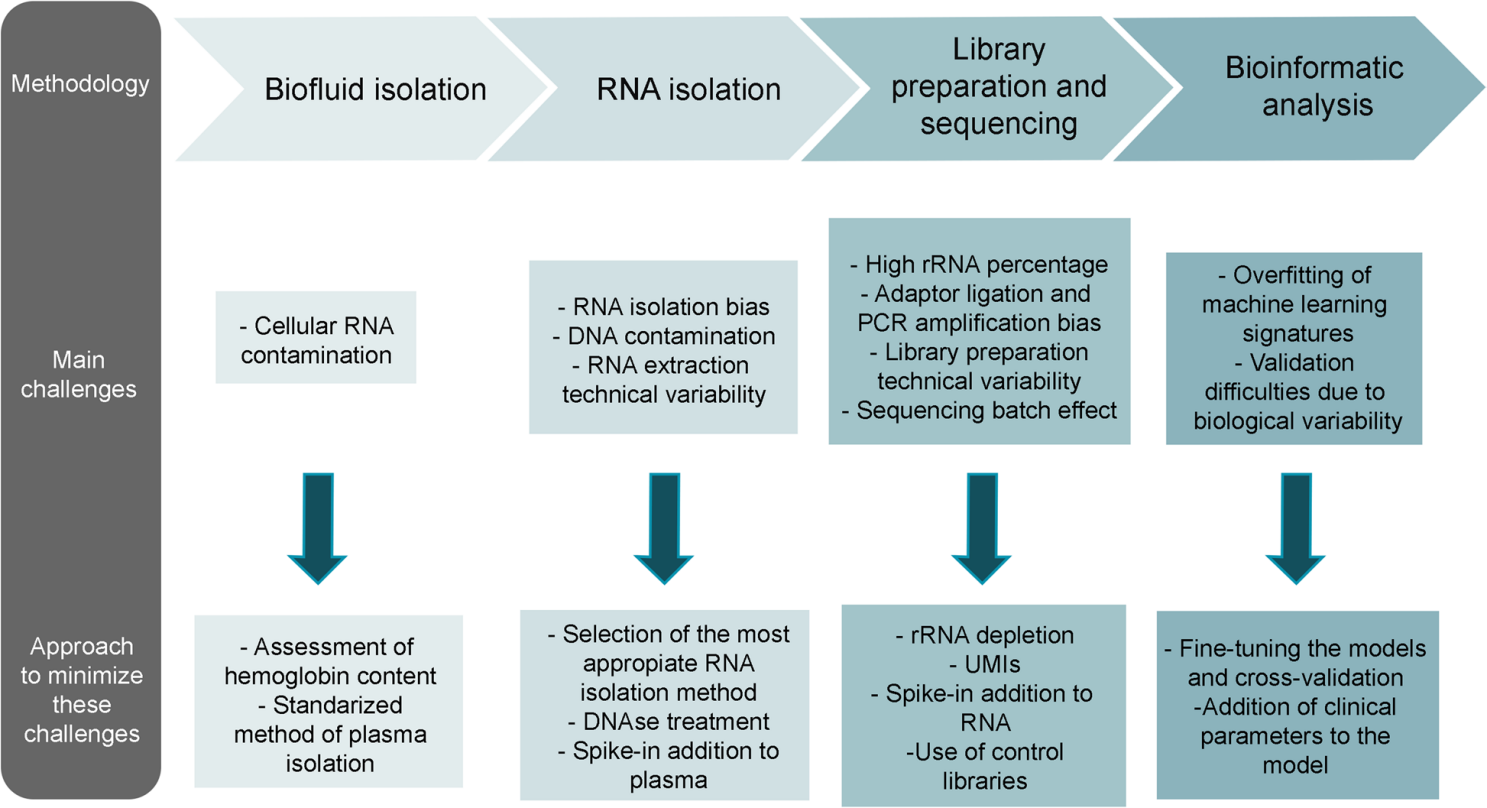
Overview of the main challenges in the process of biomarker discovery and specific steps to minimize them

Despite its promising future, the field of liquid biopsies is young and still strongly biased by technical and biological limitations. In this review, we will identify the main challenges associated with the use of cell-free long RNAs for the discovery of diagnostic biomarkers. For that purpose, we have listed the different steps involved in long RNA biomarker discovery starting from blood collection to data analysis, highlighting the main limitations associated with each step (Figs. 1 and 2).

## Plasma and serum as main biofluids

Blood carries oxygen to all organs in the body and is also the vessel of biological information shed into the circulation. This complex biofluid contains immune and blood cells, blood-clotting factors, proteins, lipoproteins, extracellular vesicles (EVs), cell fragments and nucleic acids, among other types of biomolecules [28]. In blood, cfRNA is either encapsulated inside microvesicles or forming ribonucleoprotein complexes [29]. To reduce RNA contamination from blood cells, the most common approach is to use serum (the fraction of blood remaining after the blood clotting) or plasma (the acellular fraction of the blood) instead of whole blood.

Although intrinsically different, there is limited information about which biofluid provides more biologically relevant information. Promising results have derived from both the study of serum and plasma. Even though not studied in depth, some groups reached opposite conclusions while attempting to quantify the differences associated with the analysis of serum vs plasma. For instance, Dufourd et al. suggested that circulating miRNA profiles of healthy subjects may not be affected by the type of biofluid studied [30] while others have proposed that miRNA profiles of serum and plasma are not comparable and can influence subsequent analyses [31]. An advantage of using plasma is that Klaas et al. reported higher amounts of cfRNA in plasma compared to serum [32]. Although the reason for this difference is unclear, the authors suggested that cfRNA could adhere to the blood clot during coagulation, resulting in a reduction in the cfRNA quantity.

Whether using plasma or serum, a common limitation of the study of these biofluids for biomarker discovery is the release of RNAs derived from red blood cells (RBC) and/or platelets during sample processing [33]. Regarding this, during blood clotting, a necessary step for serum isolation, RNAs are released from blood cells and platelets, affecting the circulating RNA spectrum [34, 35]. However, to separate plasma from other blood components, blood cells and platelets have to be removed with several centrifugation steps. At this point, an incorrect centrifugation or handling of the samples could result in cellular contamination [36].

In order to assess the compositional bias induced by platelet-derived EVs on the plasma transcriptome, Kim et al. [37] examined the variation of exosomes and cfRNA in human plasma due to blood processing and freeze-thaw effect. They discovered a significant reduction in the 1000-3000 nm EVs in platelet-free plasma samples, showing an ex vivo platelet EV release. Moreover, they show that while post-thaw processing reduces the amount of platelet-derived EVs, it irreversibly affects the cfRNA profile. These results suggest that banked plasma samples with different degrees of platelet removal could be incomparable.

While hemolysis controls are more common than platelet assessment, there is still a lack of consensus about the optimal steps necessary to quantify the presence of RBC RNA. In an attempt to characterize the effect of hemolysis during the study of circulating RNA biomarkers, Kirschner et al. [38] suggested the addition of a pre-analytical step to quantify RBC lysis using a spectrophotometer. According to their results, blood samples with low absorbance at 414 nm, wavelength characteristic of oxyhemoglobin [39], had similar levels of miR16, a miRNA often present in RBC and found in abundance in hemolytic samples.

Despite the absence of studies performed on cfRNA, a study found that for cellular RNA freeze-thaw cycles have a detrimental effect on the quality of the RNA obtained downstream, resulting in significantly shorter fragments [40]. Additionally, other studies have also suggested that storing plasma samples at − 80 °C leads to degradation of the nucleic acids over time [41]. This is of special interest for the study of long RNAs, which are remarkably more prone to degradation than miRNAs in plasma [21].

In summary, the obtention of plasma or serum from whole blood can result in contamination from different cellular sources, such as platelets or erythrocytes. Avoiding and assessing cellular contamination, with correct processing and quality controls, is a critical step in order to prevent and monitor the introduction of unwanted biases that could lead to wrong conclusions. In addition, avoiding freeze-thaw cycles and long-term storage improves the quality of the RNA extracted, leading to more robust and reproducible results.

## RNA isolation

The cfRNA obtained from a blood sample represents an approximation to a snapshot of the transcriptome of the individual. However, the use of different methodologies for RNA isolation leads to biases that can mask any relevant biological information.

The most common strategy for RNA isolation from biofluids is based on RNA extraction kits designed and optimized for plasma and/or serum. Commercial column-based kits are more commonly used than traditional guanidium-thiocyanate or phenol-chloroform methods. Classic methods tend to favor the isolation of selective RNA populations and often lead to reduced quantities of RNA [42]. However, the widespread use of different kits has led to intrinsic technical differences associated with kit-dependent biases. Li et al. showed that different cfRNA isolation kits yield different RNA quantities. They also found kit-dependent biases linked with the recovery of long RNAs and issues with detecting some of the most common mRNAs. Their results highlight the importance of selecting the best approach to isolate RNA depending on the end goal of the study [43].

One major concern regarding RNA isolation from plasma is DNA contamination. The majority of cfRNA isolation kits recover a fraction of the cfDNA present in the biofluid [43]. During library preparation, DNA contamination is amplified along with RNA affecting the results [44]. To minimize this bias, the most common strategy is the incorporation of an additional step where the samples are treated with DNAse. This can be done before the extraction (on-column) [45] or after [27].

In order to limit the effect of other technical biases associated with RNA isolation, some groups have proposed the addition of exogenous RNAs as controls to compare the efficiency of the RNA extraction [46, 47]. Spike-ins are exogenous RNAs with similar GC content to endogenous RNAs with sequences not found in the human genome. They are added to the samples prior to RNA isolation in a known concentration [48] and their detection is useful to assess biases introduced during RNA isolation. Spike-ins have been successfully used to compare RNA profiles across biofluids, allowing absolute quantification and revealing a 10,000 fold difference in concentration [27].

The quantity and quality of input RNA has a strong impact on downstream processes [49]. However, there is a lack of consensus on which is the best method to quantify blood-derived RNA. To date, the most commonly used methods to measure the quality and quantity of RNA are based on spectrophotometry such as Qubit [50] and Nanodrop [51]; or on capillarity, like the Bioanalyzer [52]. These methods were developed to assess the concentration of RNA in cellular samples and they are less accurate when evaluating the highly fragmented samples derived from blood. Additionally, other groups have proposed the incorporation of PCR-based methods in biomarker discovery pipelines. They use the abundance of specific genes in blood as internal controls to set a minimum concentration of RNA [53]. This approach accepts the lack of reproducible quantification methods when starting from very low input RNA and attempts only to confirm the abundance of certain genes above a threshold.

Different strategies for RNA isolation are linked to different recovery rates and to the enrichment of selective RNA types. However, several strategies are available to minimize these biases: consistency in the extraction kit used, comparison of the RNA extraction efficiency with spike-ins, assessment of samples with low RNA quality and removal of DNA contamination.

## Library preparation and sequencing

During the last decade, RNA sequencing (RNA-seq) has become the gold standard methodology for biomarker discovery. It allows the detection of known and novel transcripts and their quantification in a sample, with higher sensitivity and accuracy than other methods, such as microarrays [54]. While years ago the costs associated with this methodology were high, the drop in prices for sequencing has opened the door for the application of NGS to biomarker discovery and as a screening tool. To adapt to this new field, multiple protocols for library preparation have been developed to perform RNA-seq starting from very low RNA inputs.

Approximately 80% of the cellular RNA is ribosomal RNA (rRNA) [55], and this number is even bigger in cfRNA, with more than 90% of the RNA found in the circulation belonging to this type of RNA [45]. In order to remove high concentrations of rRNA, there are two methods: polyA enrichment or rRNA depletion. However, only the rRNA depletion step is viable in this case, due to the high fragmentation of the RNA in plasma and lack of polyA tail in most of the cfRNA molecules [56].

During library preparation, another step leading to the introduction of biases is adaptor ligation and PCR amplification. Due to secondary structures and enzyme affinity, some sequences are more prone to ligate to adaptor sequences and amplify than others [57, 58]. In order to reduce this bias, many protocols have incorporated the use of Unique Molecular Identifiers (UMIs) in the early stages of library preparation. UMIs are random sequences of 4-10 nt that are ligated to the DNA/RNA molecules before PCR amplification [59]. The use of UMIs allows the identification of PCR-duplicated reads that originate from the same initial molecule, and the in silico correction of this clonal amplification. Additionally, if the UMIs are incorporated before adaptor ligation, they can also help minimize adaptor ligation biases. When compared to traditional in silico removal of technical duplicates [60], the presence of UMIs has shown to improve reproducibility in differential gene expression analysis, especially when starting from a very low input [58, 61], as in the case of cfRNA samples.

Following the same rationale used to evaluate biases during RNA isolation, spike-ins are also useful in measuring technical variability introduced during library preparation. Similar to the spike-ins used to assess RNA isolation, they are added at a known concentration to correct for the amplification bias during library preparation [48] and can be useful to quantify and normalize in silico the sequencing output.

Finally, another possible step of bias introduction is the sequencing of the libraries. Often, batch effects are observed when samples used in the same study are sequenced in different rounds [62]. Commercially available control libraries can be added at known concentrations to measure the reproducibility of sequencing and later used to mitigate batch effects. The most common example is the PhiX Control Libraries, generated from the PhiX virus [63]. These spike-in libraries have two functions. First, they act as positive controls of the sequencing run, ensuring clustering reaction and generating a number of clusters depending on the quantity of the spike-in added. And second, as a technical control for sequencing accuracy, aligning the sequences to the reference genome of the spike-in library.

In summary, the main problems associated with library preparation and sequencing are PCR amplification and batch effects caused by technical variability. However, the use of UMI sequences and spike-ins to minimize this bias has become very prevalent in RNA-seq studies during the last years and has shown to increase the reproducibility of the results [64].

## Bioinformatics analysis

One of the most challenging steps in biomarker discovery using RNA-seq is the computational analysis of the generated data. This analysis allows to correctly interpret the molecular processes that occur in the patient. Bioinformatics analysis goes from data quality control to transcript quantification and various downstream analyses. However, since the results of the analysis are closely linked to the input quality of the data, quality assessment is a critical step in this workflow [65].

One of the most prevalent issues with the processing of raw RNA-seq data is normalization. It consists in correcting in silico for technical biases that could mask biological information [66]. RNA-seq is the most used tool to measure gene expression, although unlike other methods like RT-qPCR, it does not allow for absolute quantification. A normalization based on spike-in controls, added at the library preparation step, is one of the methods used to achieve absolute quantification of the transcriptome in cellular RNA [67]. This method is also shown to be highly reproducible in plasma samples [64]. However, some studies have found that spike-in normalization can translate to poor results due to the high variability in the spike-in amplification [68, 69]. Due to this, some groups are opting for a normalization of the samples according only to library size and gene length [45, 70]. UMI deduplication corrects for PCR biases and allows for the counting of RNA molecules in a sample, thereby improving reproducibility [61].

Once the raw data is processed, the identification of potential biomarkers can follow two different routes: comparative analysis of cfRNA profiles and machine learning (ML) methods. The former strategy is the simplest: it consists in finding genes whose expression is associated with a phenotype. This approach attempts to determine the presence of a disease or its prognosis only from the expression of a few genes. An example of this methodology is the recent study of Rasmussen et al., where they found a signature of 7 RNAs associated with preeclampsia, a condition marked by maternal hypertension that is a significant cause of maternal morbidity. This signature has a positive predictive value of 32.3%, higher than the current clinical state-of-the-art models [2]. The latter, ML-based approach, is more complex as it involves the application of ML algorithms to detect biomarker signatures predicting the likelihood of a phenotype. A ML signature combines multiple genes selected by ML algorithms and determines the presence of a disease or its prognosis. An ML-based signature normally has higher sensitivity and specificity than a signature coming from comparative analysis of cfRNA profiles [71], presumably because ML algorithms automatically assign a weight to every gene in order to maximize the accuracy of the classification. An example of this methodology is the study of Wang et al., where they found a panel of 57 RNA biomarkers that could detect COVID-19 infection with 98.1% accuracy [72].

In relatively small datasets, ML algorithms tend to generate models that fit artificially the initial cohort of samples, causing poor replication of the results in new cohorts of patients. This is especially prevalent in omics data, where the number of variables is very high [73]. This process is known as overfitting. To mitigate this, some of the most used methods are cross-validation and model simplification [74]. Cross-validation is a resampling method that uses a fraction of the data to evaluate the performance of the algorithm, whereas model simplification consists in reducing the number of genes included in the signature. This strategy of cross-validation and model simplification has already been used by multiple studies for diagnosis or prognosis of diseases.

Additionally, other research groups have proposed new approaches to enhance the reproducibility of the findings. For instance, Larson et al. [45] have focused on the study of “Dark channel” biomarkers, which are genes not expressed in non-cancer plasma, upregulated in cancer samples and detected in multiple samples to improve specificity and reduce drastically the number of false positives. Vorperian et al. [75] also proposed an interesting alternative method. They have identified the transcriptomic fingerprint of a certain number of cell types and used these profiles to deconvolute the cell types of origin of cfRNA. Using this approach it could be possible to narrow down the set of genes studied to focus only on those derived from the organ of interest, thus improving reproducibility and reducing variability.

On the one hand, the comparative analysis of cfRNA profiles is easier to implement in the clinical practice than more complex ML signatures due to its lower cost and improved practicality. On the other hand, ML signatures tend to have higher accuracy. Both methods are useful in obtaining signatures of high value for diagnosis and prognosis.

## Limitations of cfRNA biomarker discovery

The main issues limiting the applicability of liquid biopsies as screening and diagnostic tools in the clinic are technical and biological reproducibility biases. This is often linked to a lack of gold standard methods for sample processing and data analysis [76]. Due to the high technical variability observed between research groups, different studies often find unrelated RNA signatures for the same disease and type of sample. This lack of reproducibility is one of the major problems that the field is facing, with many RNA signatures entering clinical trials but none reaching the clinic so far. To try and mitigate this lack of standardization, the Global Biological Standards Institute (GBSI) published an article in 2014 to raise awareness about the lack of reproducibility and the urgent need for standards in cancer research, especially for high-throughput screening methods [77]. Since then, numerous efforts have focused on methodological standardization, such as the implementation of data repositories or the creation of reference RNA spike-in controls [76].

Besides the technical bias introduced during sample handling, external factors such as age or gender of the individual, have a strong effect on the cfRNA profile [78]. To control for these biases, every step of the study must be well controlled and documented, with balanced cohorts in all of these factors [79]. In cases where it is not possible to have balanced cohorts, these external factors should be accounted for in the statistical analysis.

A biological limitation of the study of cfRNA is that the interpersonal variability is very high [80], with some individuals showing a consistently higher expression of certain genes than others. Although their results are preliminary, they highlight the importance of a consistent normalization method to account for this biological variability.

## Current challenges in the field of RNA-based liquid biopsies

According to a recent market research study, the liquid biopsy industry is expected to exceed 5.8 billion dollars by 2026 [81], although few cfDNA tests are already in use in the clinical practice [82]. In 2020, the FDA already approved three cfDNA-based tests and, of these, Guardant Health’s Guardant360 CDx is the first one to use next generation sequencing for diagnosis [83]. Since the field of cfRNA is still young, there are no diagnostic tests based on RNA approved for the use in the clinical practice yet. There are many challenges to be addressed to translate an RNA-based liquid biopsy biomarker into the clinic, although several studies are currently undergoing clinical trials [84–88]. A robust and standardized methodology needs to be established, assessing all the possible biases that can alter the results. This will lead to more reproducible results and more robust statistical models.

One of the main challenges in the field of liquid biopsies is caused by a limitation on the number of donors in the training cohorts. Most of the studies comparing cases with controls use small retrospective cohorts to detect a disease after it is clinically reported [52, 89–91], which is suboptimal for biomarkers for early diagnosis. Only a handful of prospective studies have attempted population screening to find undiagnosed patients [92]. This approach, although optimal for diagnostic biomarker discovery and validation, requires the screening of a significant part of the population (depending on disease prevalence) and a great budgetary effort. The technical and economical requirements for such an attempt are beyond the grasp of most research centers and the biomarkers discovered in the first cohorts are not strong enough to pass an initial step of validation and attract big-pharma companies. Although multiple collaborative consortia are created to compile biological data for specific pathologies (such as the NCI Cohort Consortium for cancer), the lack of standardized methods often leads to results that highlight technical differences over biologically relevant biomarkers.

Another important aspect for the incorporation of liquid biopsies into the clinical practice is the ability of the physicians to be able to interpret the results of the biomarker model. This can be an arduous task for the medical staff that is not proficient in bioinformatics and statistical analysis. For this purpose, several research centers and companies have created cloud computing pipelines that take directly the sequencing data and generate comprehensive reports [93, 94].

## Conclusions

The field of liquid biopsies, and more specifically cell-free long RNA liquid biopsies is promising, but still young. With a relatively reduced number of studies published, there are candidate biomarkers undergoing clinical trials, but none have been approved by the regulatory agencies at the moment. In the last few years, there has been an increasing interest in liquid biopsy-based biomarkers using RNAs. However, it has been only in the last 5 years that the focus has started to switch from miRNAs to long RNAs, leading to the discovery of new disease-associated RNAs. Although there is still much work left to do to translate long cfRNA into clinical practice, a number of recent promising results suggest that long cfRNA-based liquid biopsies could be one of the next big revolutions in the field of screening and diagnosis.

## Data Availability

Not applicable.

## Abbreviations

ctDNA: Cell-free tumor DNA
cfRNA: Cell-free RNA
miRNAs: MicroRNAs
mRNAs: Messenger RNAs
lncRNAs: Long non-coding RNAs
EVs: Extracellular vesicles
RNA-seq: RNA sequencing
UMIs: Unique Molecular Identifiers
ML: Machine learning
GBSI: Global Biological Standards Institute
